# Independent Effects of HIV and Antiretroviral Therapy on the Oral Microbiome Identified by Multivariate Analyses

**DOI:** 10.1128/mbio.00409-23

**Published:** 2023-04-18

**Authors:** Clifford J. Beall, Elizabeth A. Lilly, Carolina Granada, Kelly Treas, Kenneth R. Dubois, Shahr B. Hashmi, Jose A. Vazquez, Michael E. Hagensee, Ann L. Griffen, Eugene J. Leys, Paul L. Fidel

**Affiliations:** a Division of Biosciences, The Ohio State University College of Dentistry, Columbus, Ohio, USA; b Center of Excellence in Oral and Craniofacial Biology, Louisiana State University Health Center School of Dentistry, New Orleans, Louisiana, USA; c Division of Infectious Diseases, Department of Medicine, Augusta University, Medical College of Georgia, Augusta, Georgia, USA; d Section of Infectious Diseases, Department of Medicine, Louisiana State University Health Sciences Center, New Orleans, Louisiana, USA; e Division of Pediatric Dentistry, The Ohio State University College of Dentistry, Columbus, Ohio, USA; University of Michigan—Ann Arbor

**Keywords:** oral microbiome, HIV, ART, bacteriome, mycobiome

## Abstract

The oral microbiome is an important predictor of health and disease. We recently reported significant yet modest effects of HIV under highly active antiretroviral therapy (ART) on the oral microbiome (bacterial and fungal) in a large cohort of HIV-positive (HIV^+^) and matched HIV-negative (HIV^−^) individuals. As it was unclear whether ART added to or masked further effects of HIV on the oral microbiome, the present study aimed to analyze the effects of HIV and ART independently, which also included HIV^−^ subjects on preexposure prophylaxis (PrEP) therapy. Cross-sectional analyses of the effect of HIV devoid of ART (HIV^+^ ART^−^ versus matched HIV^−^ subjects) showed a significant effect on both the bacteriome and mycobiome (*P* < 0.024) after controlling for other clinical variables (permutational multivariate analysis of variance [PERMANOVA] of Bray-Curtis dissimilarity). Cross-sectional analyses evaluating the effects of ART (HIV^+^ ART^+^ versus HIV^+^ ART^−^ subjects) revealed a significant effect on the mycobiome (*P* < 0.007) but not the bacteriome. In parallel longitudinal analyses, ART (before versus after the initiation of ART) had a significant effect on the bacteriome, but not the mycobiome, of HIV^+^ and HIV^−^ PrEP subjects (*P* < 0.005 and *P* < 0.016, respectively). These analyses also revealed significant differences in the oral microbiome and several clinical variables between HIV^−^ PrEP subjects (pre-PrEP) and the HIV-matched HIV^−^ group (*P* < 0.001). At the species level, a small number of differences in both bacterial and fungal taxa were identified within the effects of HIV and/or ART. We conclude that the effects of HIV and ART on the oral microbiome are similar to those of the clinical variables but collectively are modest overall.

## INTRODUCTION

HIV disease continues to have a worldwide health and economic impact. The advent of highly active antiretroviral therapy (ART) has greatly increased the life span and especially the quality of life of people living with HIV (PLWH) and has led to a significant reduction in opportunistic infections (1). Yet certain geographical areas where ART has not been readily/easily available (2, 3) have left many populations under continued vulnerability to a variety of oral diseases, including gingivitis, periodontitis, dental caries, endodontic infections, oropharyngeal candidiasis (OPC), oral warts, oral hairy leukoplakia, and Kaposi’s sarcoma (1, 4, 5).

The oral microbiota, which consists of more than 750 bacterial and fungal species, is considered an important component of both oral and systemic health and disease. This is exemplified by studies showing changes in the composition and diversity of the oral microbiome during periodontal disease (6–8), dental caries (6, 9), and now, more recently, HIV disease (10–19). Despite wide variations in sample sizes and methodologies, the majority of studies report a modest yet consistently significant effect of HIV (usually with ART) on the overall composition of the oral microbiome. This also includes several interesting interactions/associations between bacterial and fungal communities (11–14, 17, 20–22).

There is a multitude of clinical factors or conditions that could be significant modulators of the oral microbiome and, thus, potential confounders when examining the effects of HIV and ART (i.e., viral loads, CD4 cells, periodontal disease, caries, and cigarette smoking). Our previous publications (11, 23) detailed a number of these confounding variables that have a significant influence on the oral bacterial or fungal communities and methods for compensating for them through distance-based redundancy analysis (db-RDA). For the bacteriome, these included the presence of *Candida*, missing teeth, current cigarette smoking, periodontal disease, antimicrobials, caries status, and oral sex practices (11, 23). For the mycobiome, they include caries status, oral sex activity, and missing teeth (11, 23). While many other variables were not significant in the analysis, most of those that were significant had an effect on the microbiome that was equivalent to or often more significant than that of HIV under ART (11, 23). Interestingly, most other studies that have considered clinical variables (12, 24–27) have not incorporated them through multivariate analyses. Common to our study and others, however, is the lack of study designs to evaluate the effects of HIV and ART independently. Since most of the HIV-positive (HIV^+^) subjects were on ART and had achieved virologic control, it is quite possible that ART masked, or added to, any independent effects of HIV (e.g., immunomodulatory effects countered or added to the immunosuppressive effects of HIV). To address this, we conducted cross-sectional analyses on a group of PLWH on ART compared to a matched group who had not yet begun ART and then longitudinally monitored their initiation of ART for up to 18 months. In addition, we enrolled and longitudinally monitored a small cohort of HIV-negative (HIV^−^) subjects who were seeking preexposure prophylaxis (PrEP) with tenofovir-emtricitabine (Truvada) ART to prevent HIV infection over a similar 18-month period. Through these designs, we were able to (i) characterize the effect of HIV without ART on the oral bacteriome and mycobiome and (ii) similarly assess the independent effect of ART through analyses in the presence and absence of HIV.

## RESULTS

We recently reported on the relative abundance and diversity of the oral bacterial and fungal communities for this cohort as well as the influence of HIV under ART and a number of secondary clinical variables (11, 23). The groups analyzed in the previous publications were HIV^+^ subjects on ART and an HIV^−^ cohort matched for demographics and risk for HIV exposure (“high risk”). In the current study, we examined an additional group of HIV^+^ subjects prior to the initiation of ART and then again 1 to 6 months (early) and 6 to 18 months (late) after the initiation of therapy. In addition, a small cohort of HIV^−^ subjects who were seeking PrEP were enrolled and similarly monitored longitudinally prior to and after the initiation of PrEP with tenofovir-emtricitabine. Generally, the bacterial and fungal communities in the samples newly analyzed for this study were similar to those in our previous publications (11, 23). Subject species sample count data and accompanying clinical metadata are available in Table S1 in the supplemental material.

10.1128/mbio.00409-23.2 TABLE S1 Species counts and clinical data. Individual subject sample species data and accompanying clinical metadata are shown. Download Table S1, XLSX file, 1.4 MB. Copyright © 2023 Beall et al. 2023 Beall et al. https://creativecommons.org/licenses/by/4.0/ This content is distributed under the terms of the Creative Commons Attribution 4.0 International license.

In the longitudinal cohort, ART was defined as effective when the quantitative viral load was <1,000 copies/mL (Fig. S1). The median viral load pre-ART was 43,685 copies/mL, whereas the median after at least 100 days on ART was <20 copies/mL (*P* = 0.0002). The pre-ART median CD4 count was 426 cells per mm^3^, and this was increased to 525 cells per mm^3^ after at least 100 days on ART (*P* = 0.0002). These results indicated that ART was effective with good compliance.

10.1128/mbio.00409-23.1 FIG S1 ART greatly decreased viral loads and modestly increased CD4 counts. The left side shows box-and-whisker plots of CD4 counts from HIV^+^ study subjects undergoing ART, and the right side shows viral loads from HIV^+^ subjects. The limit of detection of the assay for the viral load is 20 copies per mL. Download FIG S1, TIF file, 2.4 MB. Copyright © 2023 Beall et al. 2023 Beall et al. https://creativecommons.org/licenses/by/4.0/ This content is distributed under the terms of the Creative Commons Attribution 4.0 International license.

**FIG 1:**
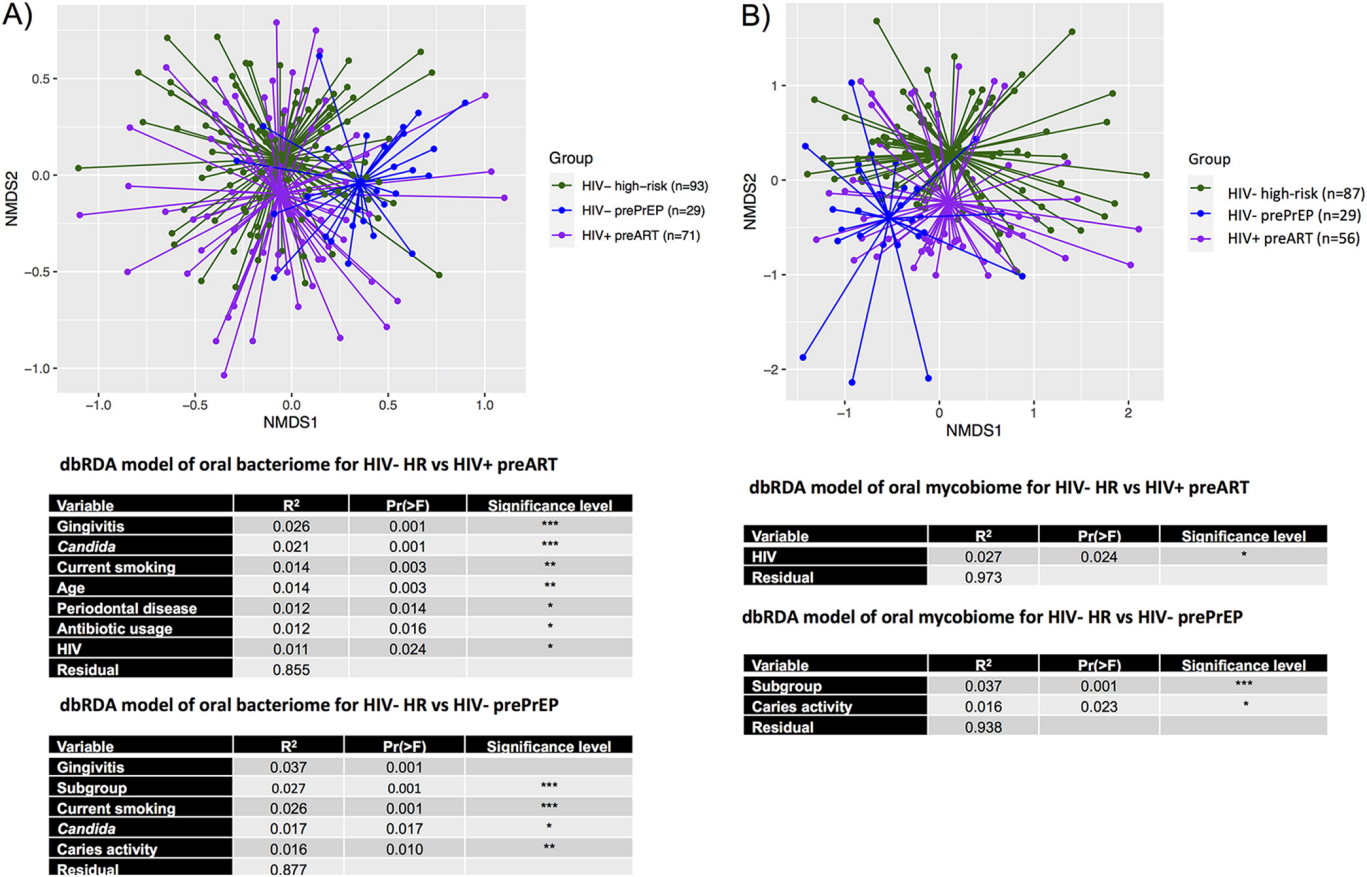
Multivariate distance-based analysis of the effects of HIV on the oral bacterial community composition. Nonmetric multidimensional scaling ordination of Bray-Curtis dissimilarities between oral bacterial (A) and fungal (B) communities is shown for HIV^−^ subjects (high risk [HR]), HIV^+^ subjects prior to the initiation of ART, and HIV^−^ pre-PrEP subjects. The spiders connect the sample points to the centroid of each group. The accompanying table indicates PERMANOVA of the significant differences between HIV^−^ and HIV^+^ pre-ART groups in multivariate analyses. *, *P* < 0.05; **, *P* < 0.01; ***, *P* < 0.001.

Figure 1 shows the independent effects of HIV on oral bacterial and fungal communities via nonmetric multidimensional scaling (NMDS) based on Bray-Curtis dissimilarities in HIV^+^ subjects prior to the start of ART (pre-ART), matched HIV^−^ high-risk subjects, and HIV^−^ subjects prior to PrEP (pre-PrEP) (all baseline visits). Multivariate analyses of the oral bacteriome revealed significant differences contributed by several clinical variables, including gingivitis, the presence of *Candida*, current cigarette smoking, age, periodontal disease, antibiotic usage, and HIV (Fig. 1A). These influences accounted for ~14% of the variance in the oral bacteriome, whereas HIV accounted for only 1.1% of the identified variance (*P* < 0.024). From the position of the centroids in the NMDS analysis, it is apparent that the HIV^−^ pre-PrEP subjects have different bacterial communities than both the HIV^−^ high-risk and HIV^+^ pre-ART groups (*P* < 0.001). These differences are partially explained by demographic/clinical variables, including less cigarette smoking, less caries, and less gingivitis in the HIV^−^ pre-PrEP group.

When evaluating the oral mycobiome, HIV was considered the primary influence on the community variance (2.7%) when comparing HIV^+^ pre-ART and HIV^−^ high-risk subjects (*P* < 0.024) (Fig. 1B). No other clinical variables were significant contributors. When considering the two populations of HIV^−^ subjects, the distance between the centroids indicates that the oral mycobiomes in the pre-PrEP and HIV^−^ high-risk groups are also distinct (*P* < 0.001), with the presence of caries being identified as a major contributor (*P* < 0.023).

**FIG 2:**
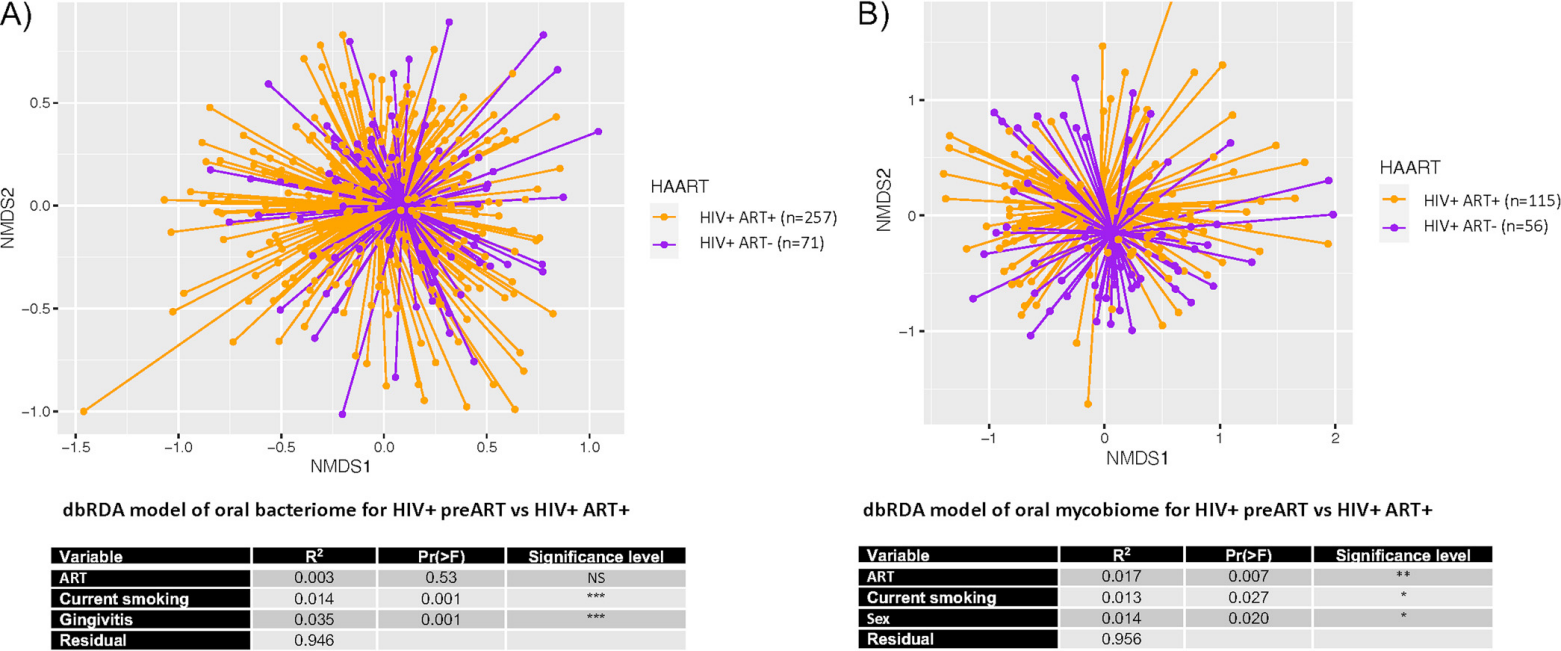
Cross-sectional analysis of the effect of ART on the microbiome in HIV^+^ individuals. HIV^+^ subjects enrolled under ART were compared to HIV^+^ subjects prior to the initiation of ART. Nonmetric multidimensional scaling ordination of Bray-Curtis dissimilarities between oral bacterial (A) and fungal (B) communities is shown. The spiders connect the sample points to the centroid of each group. The accompanying table indicates PERMANOVA of the significant differences between groups in multivariate analyses. HAART, highly active antiretroviral therapy; *, *P* < 0.05, **, *P* < 0.01, ***, *P* < 0.001; NS, not significant.

Figure 2 shows the NMDS ordination of Bray-Curtis dissimilarities for the independent effects of ART on the oral bacteriome and mycobiome using a cross-sectional comparison of two separate groups of HIV^+^ subjects, HIV^+^ ART^+^ subjects (baseline visit) and HIV^+^ ART^−^ subjects (baseline visit prior to the initiation of ART). Multivariate analyses of the oral bacteriome did not demonstrate any significant effect of ART but revealed significant contributions of gingivitis and current smoking (*P* < 0.001) (Fig. 2A). Multivariate analyses of the oral mycobiome showed a modest effect of ART (*P* < 0.007) along with lesser contributions from current smoking and oral sex activity (*P* < 0.027 and *P* < 0.02, respectively) (Fig. 2B).

**FIG 3:**
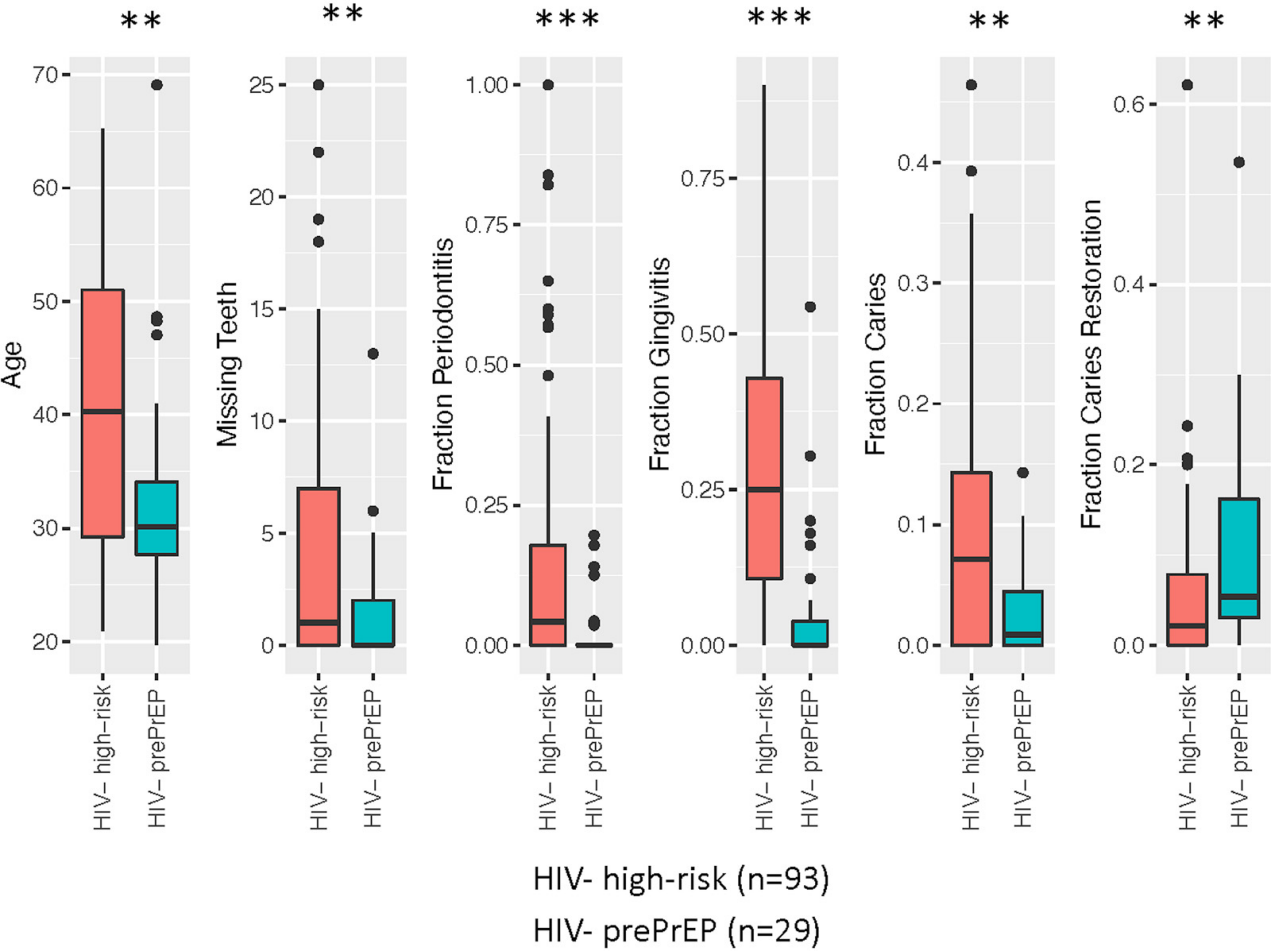
Comparison of ordinal clinical variables between HIV^−^ high-risk subjects and HIV^−^ pre-PrEP subjects. Box-and-whisker plots are shown for the indicated variables, with the boxes showing the 25th, 50th, and 75th quartiles; the whiskers showing 1.5 times the interquartile difference or the limit of the data if lower; and outliers shown as points. Significance is indicated with asterisks (**, *P* < 0.01; ***, *P* < 0.001).

The differences between the HIV^−^ high-risk subjects and the HIV^−^ pre-PrEP subjects were further assessed by evaluating general and oral health parameters as continuous variables. The results in Fig. 3 show that the HIV^−^ high-risk group was significantly older (*P* < 0.01), had significantly fewer teeth (*P* < 0.01), and had higher incidences of periodontitis, gingivitis, and caries (*P* < 0.001). The HIV^−^ pre-PrEP subjects had higher levels of caries restorations (*P* < 0.01). Other significant differences in the HIV^−^ PrEP group included higher numbers of male and white subjects and higher proportions of alcohol consumption (*P* < 0.001), marijuana use (*P* < 0.01), and oral sex activity (*P* < 0.001) but a lower proportion of cigarette smoking (*P* < 0.01).

**FIG 4:**
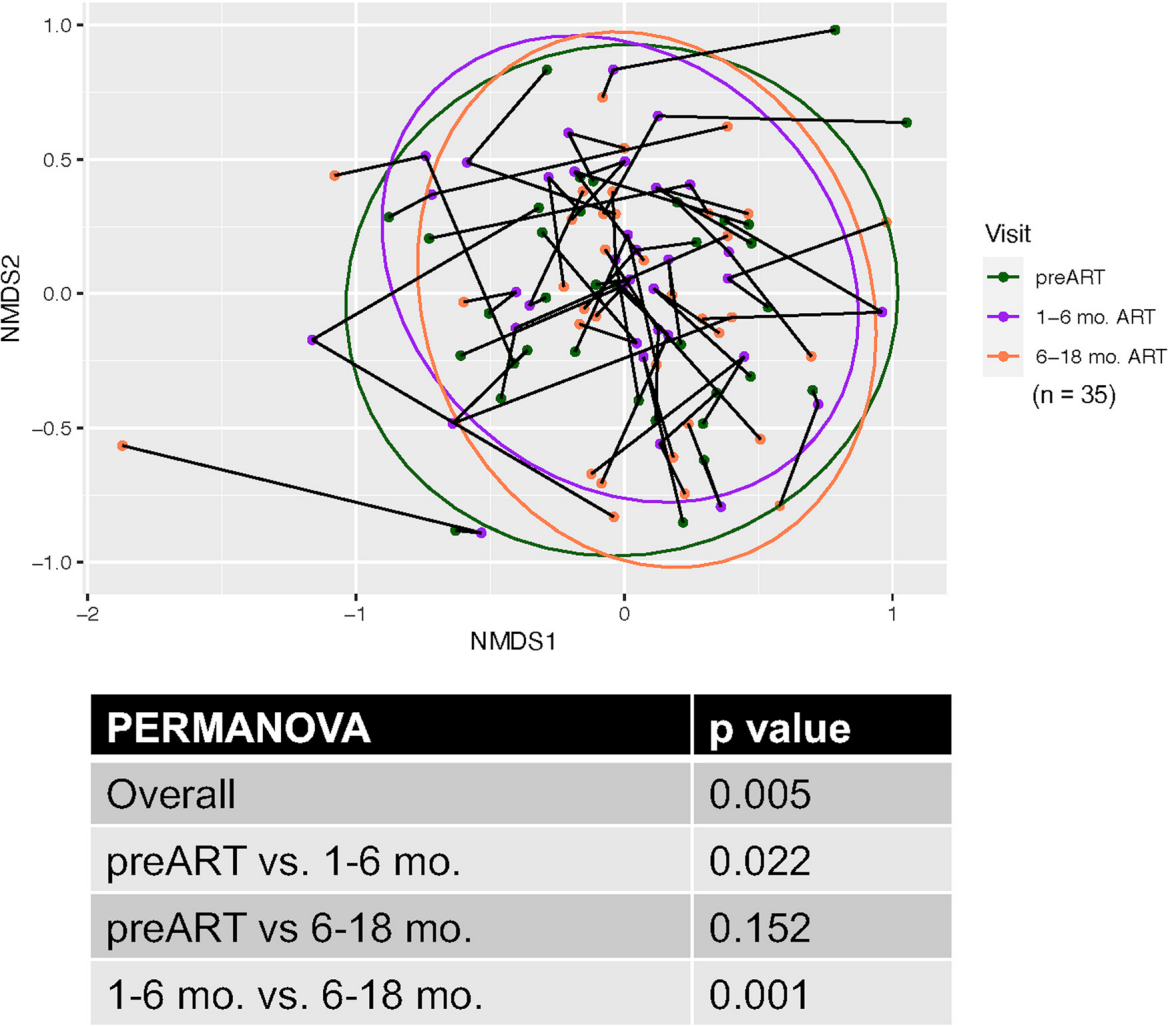
Longitudinal analysis of the effect of ART on the oral bacteriome of HIV^+^ individuals. ART was initiated following HIV diagnosis. Samples were taken preinitiation and 1 to 6 months and 6 to 18 months post-ART. Nonmetric multidimensional scaling ordination of the Bray-Curtis dissimilarity between oral bacterial communities is shown. The ellipses represent the 95% confidence region for the centroid of the group. Line segments connect the three NMDS ordination points from each subject that were collected over time. The accompanying table shows the PERMANOVA comparisons overall and *post hoc* pairwise comparisons between time points.

Figure 4 shows the longitudinal effect of ART on the oral bacteriome for a set of 35 HIV^+^ subjects who were each sampled at 3 visits, the baseline visit prior to ART and 2 visits after the initiation of ART (1 to 6 months and 6 to 18 months). The results show a modest but significant effect overall of ART (*P* < 0.005), accounting for a 2.5% change in the bacterial composition overall. In the NMDS ordination, the clustering and nonlinearity of the line segments suggest that samples from the same subject over time are more similar than those from different subjects. This was confirmed by permutational multivariate analysis of variance (PERMANOVA) using subject as a variable (*R*^2^ = 0.58; *P* < 0.001). The nested analyses showed that the significant differences were primarily between baseline and 1 to 6 months post-ART (*P* < 0.022); comparisons between baseline and 6 to 18 months post-ART were not significant, while comparisons between 1 and 6 months and 6 to 18 months post-ART were significant (*P* < 0.001).

**FIG 5:**
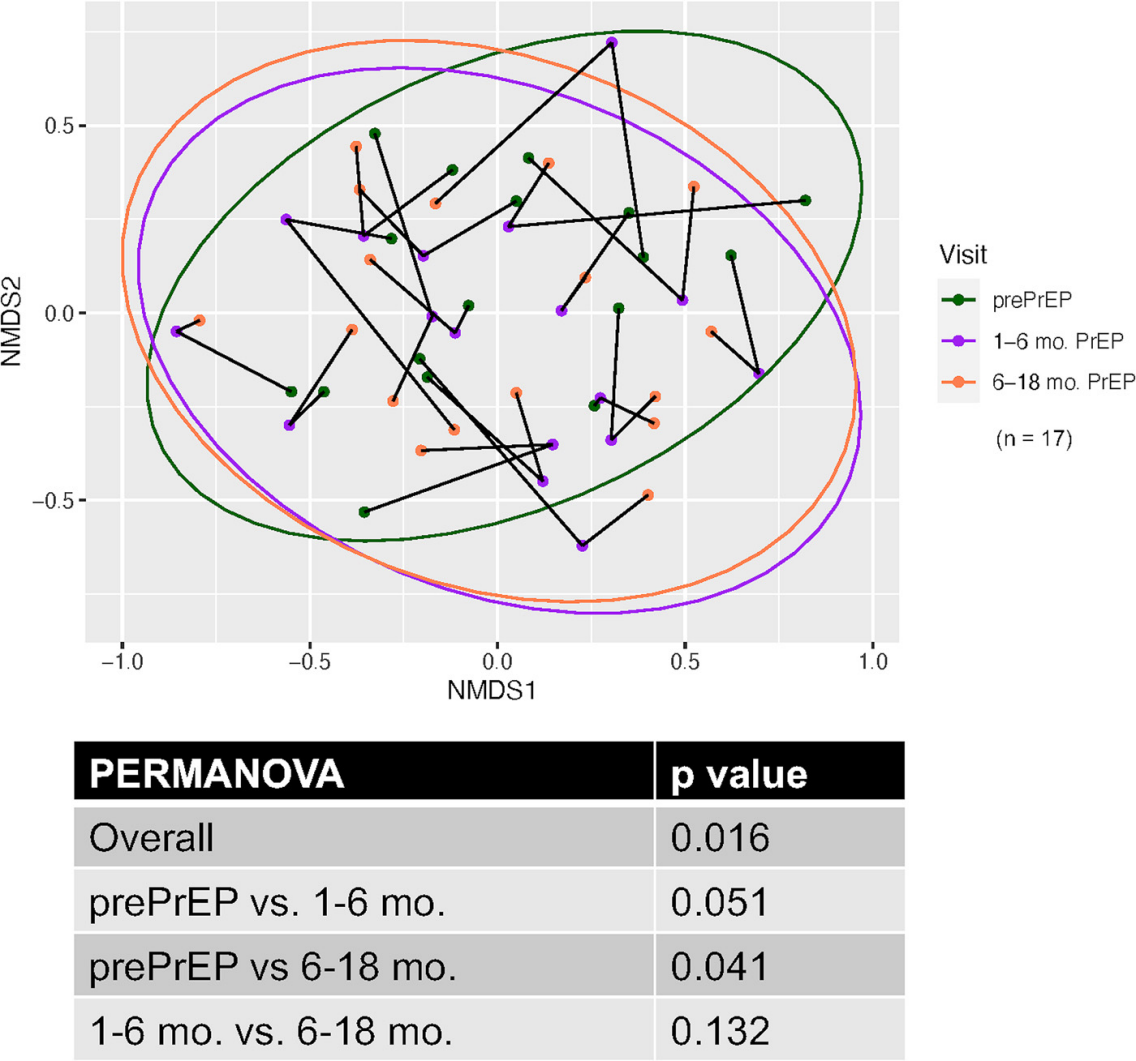
Longitudinal analysis of the effect of ART on the oral bacteriome in HIV^−^ PrEP subjects. ART was initiated upon request for HIV-negative subjects under the PrEP program. Samples were taken prior to the initiation of ART (Pre-PrEP) and 1 to 6 months and 6 to 18 months after the initiation of ART. Nonmetric multidimensional scaling ordination of Bray-Curtis dissimilarities between oral bacterial communities is shown. Ellipses represent the 95% confidence regions of the group centroids. Line segments connect the three NMDS ordination points from each subject that were collected over time. The accompanying table shows the PERMANOVA comparisons between all time points.

Figure 5 shows the longitudinal effect of ART/tenofovir-emtricitabine on the oral bacteriome in a set of 17 HIV^−^ PrEP subjects sampled similarly at baseline and 1 to 6 months and 6 to 18 months after the initiation of tenofovir-emtricitabine. The results show a similar modest, but significant, overall effect of tenofovir-emtricitabine (*P* < 0.016), accounting for a 3.4% change in the bacterial composition. The nested analyses showed a significant difference between baseline and 6 to 18 months post-ART (*P* < 0.041), with a trend toward significance between baseline and 1 to 6 months post-ART (*P* < 0.051). As with the HIV^+^ longitudinal group, the NMDS ordination of the HIV^−^ PrEP group shows a similar clustering of samples from individuals, which was confirmed by PERMANOVA with subject as the variable (*R*^2^ = 0.65; *P* < 0.001).

**FIG 6:**
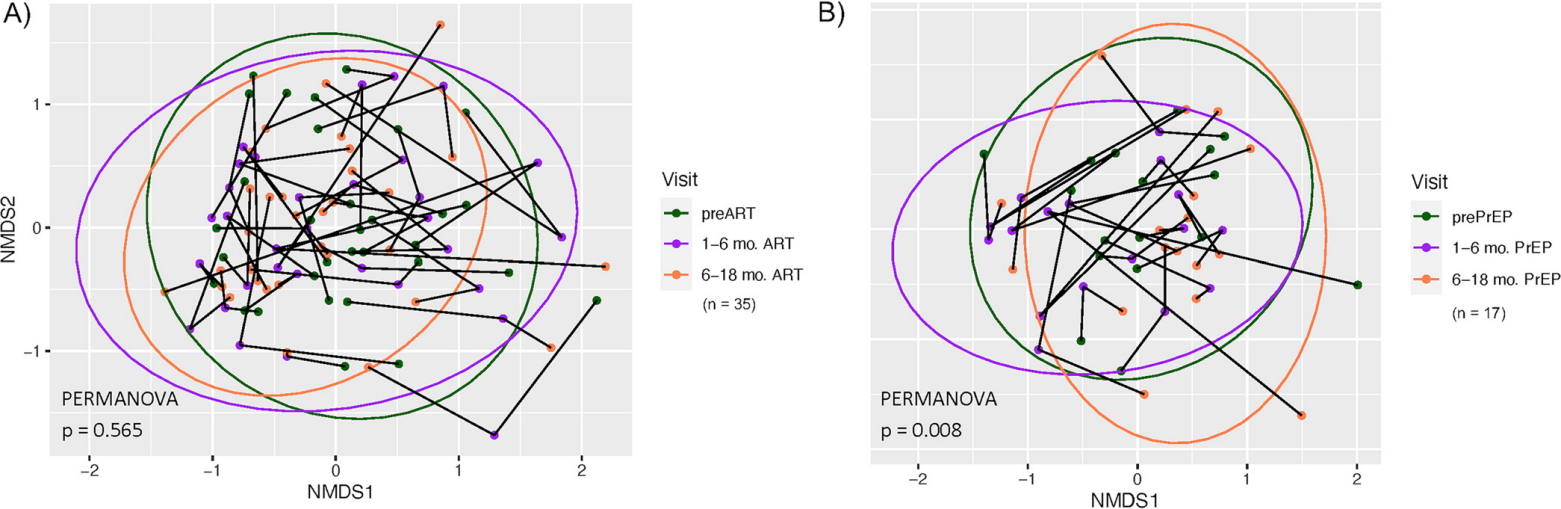
Longitudinal analysis of the effect of ART on the oral mycobiome in HIV^+^ and HIV^−^ PrEP individuals. ART was initiated following HIV diagnosis for HIV^+^ subjects and upon request for HIV^−^ subjects under the PrEP program. Samples were taken prior to the initiation of ART/tenofovir-emtricitabine (prePrEP) and 1 to 6 months and 6 to 18 months after the initiation of therapy. Nonmetric multidimensional scaling ordination of Bray-Curtis dissimilarities between oral fungal communities is shown. The dissimilarities for fungal communities were calculated using qPCR-adjusted abundances. The ellipses represent the 95% confidence intervals for the position of the group centroid. Line segments connect the three NMDS ordination points from each subject that were collected over time. The overall PERMANOVA *P* values for each longitudinal experiment are shown.

Figure 6 shows the longitudinal effect of ART/tenofovir-emtricitabine on the oral mycobiome in both HIV^+^ and HIV^−^ PrEP subjects before and after the initiation of therapy. The results showed no significant differences between baseline and 1 to 6 or 6 to 18 months post-ART/tenofovir-emtricitabine therapy. Based on the positioning of samples from the same subjects and the lines connecting them, there appears to be less clustering of samples from the same subject for the fungal communities than for the bacterial communities, and this was confirmed by the lower *R*^2^ value derived by PERMANOVA with subject as a variable, although it was still significant (*R*^2^ = 0.020; *P* = 0.002).

To extend the analysis, negative binomial linear models were used to evaluate changes in species-level taxa. The analysis was performed for three situations: the two longitudinal cohorts prior to and then during antiretroviral treatments to evaluate the effect of drug therapy on individual taxa, and the cross-sectional comparison between the HIV^+^ pre-ART and HIV^−^ high-risk groups to examine the role of HIV without drug therapy. For the HIV^+^ longitudinal group, none of the bacterial or fungal taxa were significantly different after adjustment for multiple testing. For the HIV^−^ longitudinal group, no fungal taxa were significant, but 4 bacterial taxa were increased with drug prophylaxis: the Streptococcus salivarius species group, Fusobacterium nucleatum subsp. *fusiforme*, Prevotella denticola, and Catonella morbi. In the cross-sectional comparison between HIV^+^ pre-ART and matched HIV^−^ groups (effect of HIV alone), the abundances of the bacterium Porphyromonas pasteri and the fungus Candida dubliniensis were each lower in the HIV^+^ group.

## DISCUSSION

We previously reported on the characteristics of the oral bacterial and fungal communities in a group of HIV^+^ subjects receiving ART and a demographically matched group of HIV^−^ subjects using whole-mouth gargle/rinse sampling (11, 23). The primary conclusions were that multiple clinical factors have a significant influence on the oral bacterial and fungal community composition and diversity, including the presence of HIV under ART. However, the impact of HIV/ART was much smaller than expected. An important question in these analyses was whether ART was adding to or masking a more significant effect of HIV on the oral microbiome. We addressed this in the current study by extending the analyses in two ways that enabled the independent evaluation of the effects of HIV and components of ART. One was the recruitment of a group of HIV^+^ subjects prior to the initiation of ART, which allowed a cross-sectional comparison to HIV^+^ subjects on ART (the group that we studied in the previous reports). The second approach included longitudinal analyses of the HIV^+^ subjects pre- and post-ART initiation, together with a similar design with a group of HIV^−^ PrEP subjects who initiated a specific form of ART, namely, tenofovir-emtricitabine, for HIV transmission prophylaxis. Due to attrition in these longitudinal studies, these groups were smaller than the cross-sectional groups but have the important advantage of case-control analyses with more limiting confounding variables.

The evaluation of the oral bacterial and fungal communities in the HIV^+^ pre-ART group compared to the HIV^−^ high-risk controls (to measure the independent effect of HIV) showed that HIV had a significant effect on the oral microbiota (*P* = 0.024 in both cases), although the effect was considered modest by the *R*^2^ values (1 to 3% variance). Of note, this cohort of PLWH is relatively immunocompetent (see Fig. S1 in the supplemental material). For the bacteriome, the percent variance was generally lower than that contributed by other clinical variables such as gingivitis, *Candida* positivity, current smoking, age, periodontal disease status, and antibiotic usage. On the other hand, HIV was the only significant contributing variable for the mycobiome. Thus, similar to the analyses of HIV^+^ ART^+^ subjects (11, 23), the overall effect of HIV devoid of ART on the bacteriome and mycobiome was modest but significant. We interpret these results as indicating that the presence of ART in the original analysis was not adding to or masking a greater effect of HIV. Based on these results, we hypothesized that the independent effects of ART on the oral bacteriome and mycobiome would similarly be relatively modest.

The independent effect of ART on the oral microbiome was indeed modest. This was first shown by the cross-sectional oral bacteriome and mycobiome analyses comparing HIV^+^ subjects in the presence and absence of ART. In fact, in the cross-sectional bacteriome analysis, the presence of ART failed to show any significant effect despite significant effects contributed by two clinical variables (gingivitis and smoking). For the mycobiome, the effect of ART, although again modest, was greater than that of the significant contributions by the clinical variables, which included smoking and oral sex practices. The longitudinal analyses similarly showed minor effects of ART but with some disparity with the cross-sectional analyses: the significant effects shown for the oral bacteriome were limited to early times (1 to 6 months) post-ART, whereas no effects were shown for the mycobiome. These differences are likely due to several factors. The cross-sectional analyses included a larger sample size and, therefore, more statistical power but with the potential for more confounding variables. The longitudinal analyses, as a case-control design, had much less of an influence from confounding variables but were limited by the sample size. Regardless of the disparities, however, and possibly more of a reflection of the disparate results, the effect of ART on the oral bacteriome and mycobiome can be considered fairly minimal.

Another measure of the effects of ART was the influence of tenofovir and emtricitabine, two common components of ART regimens, in the longitudinal analysis of the HIV^−^ PrEP subjects. Here again, the effects of this antiretroviral therapy (Truvada) on both the oral bacteriome and mycobiome were minimal. Hence, these results continue to support the hypothesis that the effect of ART on the oral microbiome is minimal. PrEP subjects represent an important HIV^−^ group to evaluate the effects of ART. While a limited number of reports have begun to evaluate the effect of PrEP on vaginal and rectal microbiomes as a means of assessing any dysbiosis in HIV transmission (28, 29), this is the first report on the oral microbiome in PrEP subjects.

The effects of clinical variables on the oral microbiome in HIV^−^ and HIV^+^ subjects in the presence or absence of ART were consistent with those that we reported previously (11, 23), suggesting that the contributions of the clinical variables are fairly constant over time while patients are on ART. These include both continuous and categorical variables. Variables that did change on ART included increased CD4 T cells (*P* < 0.0002) and decreased HIV loads (*P* < 0.00002), indicative of “effective” ART. We reported previously that varying CD4 counts and HIV loads had no appreciable effects on the oral microbiome (11, 23).

There have been only a limited number of studies that have evaluated the effects of ART on the oral microbiome longitudinally in HIV^+^ subjects (25, 30) and none to date in HIV^−^ subjects. Similar to our observations, those previous longitudinal studies (predominantly 6 months post-ART) showed relatively minor differences. In one study, the effects of ART correlated primarily with the immune status of the subjects (25). In the other study, the effects of ART on the gut microbiome were more significant than the effects on the oral microbiome (30). Unfortunately, those studies did not include an HIV^−^ control group for the converse analysis of the independent effects of HIV. While our analysis was overall more comprehensive, with the multivariate analysis of clinical confounders and the inclusion of PrEP subjects, all of these studies are generally in consensus that ART has relatively minor effects on the oral microbiome. This is also supported by the relatively small number of changes in species for the effects of HIV or ART. In fact, of the top 50 bacterial taxa and 25 fungal taxa identified, the effects of HIV resulted in changes in only a single bacterial taxon (Porphyromonas pasteri) and a single fungal taxon (Candida dubliniensis), both of which were reduced in those with HIV. A reduction in C. dubliniensis was also shown previously for HIV^+^ subjects under ART compared to a matched HIV^−^ group (11, 23). For the effects of ART, the only changes identified included 4 bacterial taxa that were increased in HIV^−^ subjects taking PrEP.

In the longitudinal cohorts, there was a relatively high degree of similarity of samples from the same subject compared to samples from different subjects. This stability of the oral microbiome has been noted in other studies (31, 32). The degree of similarity within subjects was especially notable for bacterial communities, where the *R*^2^ values indicate that over half of the variation between samples could be attributed to the subject variable. In the case of fungal communities, the smaller variations attributed to the subject (lower *R*^2^ values) are likely due to the lower diversity of oral fungi, with fewer species, and the tendency for subjects to independently harbor as few as two to three dominant species.

The HIV^−^ groups (high risk versus PrEP) were clearly distinct based on both the oral microbiome composition and the status of clinical variables. Specifically, in addition to a distinct oral microbiome composition, the HIV^−^ high-risk group had more gingivitis, a higher prevalence of smoking, a greater presence of *Candida*, more caries, and greater numbers of missing teeth than the PrEP subjects. Oral hygiene behaviors, on the other hand, were fairly similar between the two groups, including the frequencies of teeth cleaning, flossing, brushing, and mouthwash use. Thus, despite similar oral hygiene behaviors, and although the sample size for the PrEP subjects is small, they appear to be in better oral health than the HIV^−^ high-risk group. The demographic makeup of the cohorts was different as well, with the HIV^−^ PrEP subjects consisting predominantly of younger white males, compared to predominantly older black males in the HIV^−^ high-risk group. In terms of recreational behaviors, the HIV^−^ PrEP subjects used alcohol and marijuana more frequently and had more oral sex but were less likely to smoke and drink carbonated beverages. Some differences in behaviors may be reflective of the PrEP subjects being in a monogamous relationship albeit with an HIV^+^ partner (requirement for participation in PrEP). Other differences may be attributed to the age gap between the two groups, which was ~10 years.

In conclusion, this study represents a robust comprehensive analysis of the independent effects of HIV and ART on the oral microbiome, both bacterial and fungal, in a large cohort of HIV^+^ and HIV^−^ subjects that included cross-sectional and longitudinal analyses. Overall, the independent effects of HIV and ART on the oral microbiome are significant and similar to those of the clinical variables but collectively modest, consistent with our previous results with HIV^+^ subjects on ART (11, 23). Although the study was comprehensive, with a large sample size, there were several limitations as well. These included the multiple enrollment and collection sites (personnel, geographical location, and physical space), which, while offering diversity, was repeatedly identified as a confounding factor influencing differences in the oral microbiome (11, 23). Another limitation of the longitudinal analysis was the dropout rate of almost 50%, which reduced the power of the longitudinal analysis. Finally, the amplicon sequencing approach used allows identification only to the near-species level. Hence, more specific differences between bacterial and fungal strains may have been missed. Nevertheless, within the limitations of the study, the contributions of HIV, ART, and the clinical variables evaluated accounted for 15% of the total change in the oral bacteriome/mycobiome via comparative analyses within the cohort. Thus, the remaining 85% of the total variance in the oral bacteriome or mycobiome composition is unaccounted for (residual). It will be important to conduct additional studies to determine what additional nondental factors (e.g., genetics, diet, environment, socioeconomic, and stochastic effects) contribute to the overall variance in the oral microbial composition within the cohort.

## MATERIALS AND METHODS

### Clinical methods.

Recruitment, clinical data collection, asymptomatic yeast detection, diagnosis of OPC, and oral rinse sample collection were carried out as previously described (23). The original cohort (23) consisted of 470 subjects, *viz*., 121 HIV^−^ and 349 HIV^+^ subjects. The previous bacteriome analyses included 257 HIV^+^ subjects on ART and 89 matched HIV^−^ high-risk subjects (23). The previous mycobiome analyses included 149 HIV^+^ subjects on ART and 88 matched HIV^−^ subjects (11). In the current study, HIV^+^ subjects also included those enrolled and evaluated prior to ART (*n* = 71) (54 male and 17 female patients; 55 black, 10 white, and 6 of an unspecified race; and median age of 40.7 years) and those enrolled on ART and evaluated previously (*n* = 257) (23). Also included were matched HIV^−^ subjects who were at high risk for HIV exposure based on behaviors (*n* = 93) (23). The HIV^+^ patients enrolled prior to ART were placed on ART after baseline evaluation/testing and monitored longitudinally for up to 18 months, with samples being collected between 1 and 6 months (early) (*n* = 58) and again between 6 and 18 months (late) (*n* = 42). Antiretroviral regimens were categorized by the mechanism of action and included reverse transcriptase inhibitors (RTIs), integrase inhibitors, nonnucleotide reverse transcriptase inhibitors (NNRTIs), and receptor antagonists. For those enrolled while on ART, most were taking one of 3 regimens: RTIs alone, RTIs with the addition of integrase inhibitors, or RTIs with the addition of NNRTIs. No significant differences in microbial community compositions were observed among any of these regimens (data not shown). In addition, a group of HIV^−^ subjects who were placed on PrEP (*n* = 29) as part of an established program intended to prevent HIV transmission in HIV^−^ subjects who were with HIV^+^ partners (26 male and 3 female patients; 19 white, 8 black, and 2 of an unspecified race; and median age of 30.1 years). PrEP subjects were monitored similarly, with early (*n* = 21) and late (*n* = 17) evaluations after therapy that included tenofovir and emtricitabine (Truvada). The mycobiome analysis included a proportion of those in the bacteriome analyses, consisting of 56 HIV^+^ subjects and 29 HIV^−^ subjects placed on ART/tenofovir-emtricitabine, 87 matched HIV^−^ subjects, and 115 HIV^+^ subjects under ART. Analyses were conducted only on subjects with complete data sets. For HIV^+^ subjects, the final longitudinal analyses post-ART included 35 subjects. For HIV^−^ PrEP subjects, the final longitudinal analyses included 17 subjects.

### DNA extraction and sequencing.

DNA was prepared from whole-mouth gargle/rinse samples as described previously (11, 23, 33), with variations based on whether it was to be used for bacterial or fungal sequencing.

Sequencing libraries were prepared by two-step procedures based on the Illumina 16S protocol (11, 23, 34), with index sequences added in a second amplification step. The set of dual indices was originally developed by Kozich et al. (35). At a minimum, one no-template control and one mock community control were included on each plate. The mock community samples universally gave the sequences expected from the genomes included, while the no-template controls typically gave small numbers of random sequences.

### Sequence data processing.

Sequence reads were combined into contigs using mothur and aligned to the CORE database using BLASTn. Further processing was carried out using python and php scripts as described previously (11, 36–38).

### qPCR for total bacterial and fungal DNAs.

The concentration of bacterial DNA was quantitated using a Bio-Rad iCycler real-time detection system. One microliter of DNA was included in a 20-μL reaction mixture with 1× SsoFast EvaGreen supermix (Bio-Rad) and primers Eub338F (ACT CCT ACG GGA GGC AGC AG) and Eub518R (ATT ACC GCG GCT GCT GG). The thermal steps were 98°C for 2 min followed by 45 cycles of 98°C for 5 s and 64°C for 5 s. Purified Porphyromonas gingivalis genomic DNA was used as a standard.

Fungal DNA was measured by quantitative PCR (qPCR) of the ITS2 (internal transcribed spacer 2) region. The primers used were 5.8s-F (TCG ATG AAG ARC GCA GC) and the 28S reverse primer that was used for sequencing. The cycling conditions and instrument settings were the same as those used for bacterial qPCR. Purified Candida albicans genomic DNA was used as the standard.

### Statistical analysis.

Clinical data were collected and calculated as described previously (11, 23). Nonmetric multidimensional scaling of all possible pairwise Bray-Curtis dissimilarities was performed using the metaMDS function of the vegan package in R. For confounder analysis, the independent contributions of clinical variables to the microbial community composition were determined by PERMANOVA, a nonparametric permutation-based analysis of variance (ANOVA) (adonis function in vegan).

Distance-based redundancy analysis (db-RDA) was done using capscale (vegan). Variables were included in a stepwise db-RDA model selection process (ordiR2step; vegan). The marginal significance of the remaining variables was tested by a permutation-based ANOVA for constrained ordinations (anova.cca; vegan). The statistical test was done on the marginal effect of HIV in the presence of the other significant variables, avoiding issues with possible correlations between variables. Only the clinical variables that were significant contributors to the variation in the microbial community composition after adjusting for multiple comparisons (39) were entered into the model selection for the db-RDA ordination. In the case of the longitudinal data sets, the strata parameter of the adonis function in vegan was set to the subject identifier to restrict the permutations appropriately to adjust for within-subject similarity.

Statistically significant changes in bacterial taxa were determined using negative binomial linear models. For the case of the longitudinal studies on ART/PrEP, subject was used as a random effect in a mixed-effects analysis. The models were implemented using the glmmTMB library in R (40). For bacterial populations, the top 50 species-level taxa with the highest relative abundances were included, while for fungal communities, the top 25 were included. The models included all measured clinical variables that had a significant influence on the entire communities in the same subject groups. Significant taxa were selected by *q* values of <0.05 following adjustment with the Benjamini-Hochberg false discovery rate procedure (39).

To determine the statistical significance of changes in CD4 counts and HIV loads following ART while accounting for repeated measurements, we selected pairs of measurements from subjects pre-ART and post-ART (*n* = 28 for CD4 counts and *n* = 26 for HIV loads) and performed a paired Wilcoxon test (using the wilcox.test function of the stats package in R).

The code used in the statistical analyses has been deposited at the OSU code repository at https://code.osu.edu/beall-3/hiv_longitudinal.

### Data availability.

The sequence files generated in this study have been deposited in the NCBI Sequence Read Archive (SRA) (22) under BioProject accession number PRJNA530161.
