# Supplementary figures and images for: Independent Effects of HIV and Antiretroviral Therapy on the Oral Microbiome Identified by Multivariate Analyses
Source: mBio. 2023 Apr 18;14(3):e00409-23. doi: 10.1128/mbio.00409-23 (PMC10294613; doi:10.1128/mbio.00409-23)

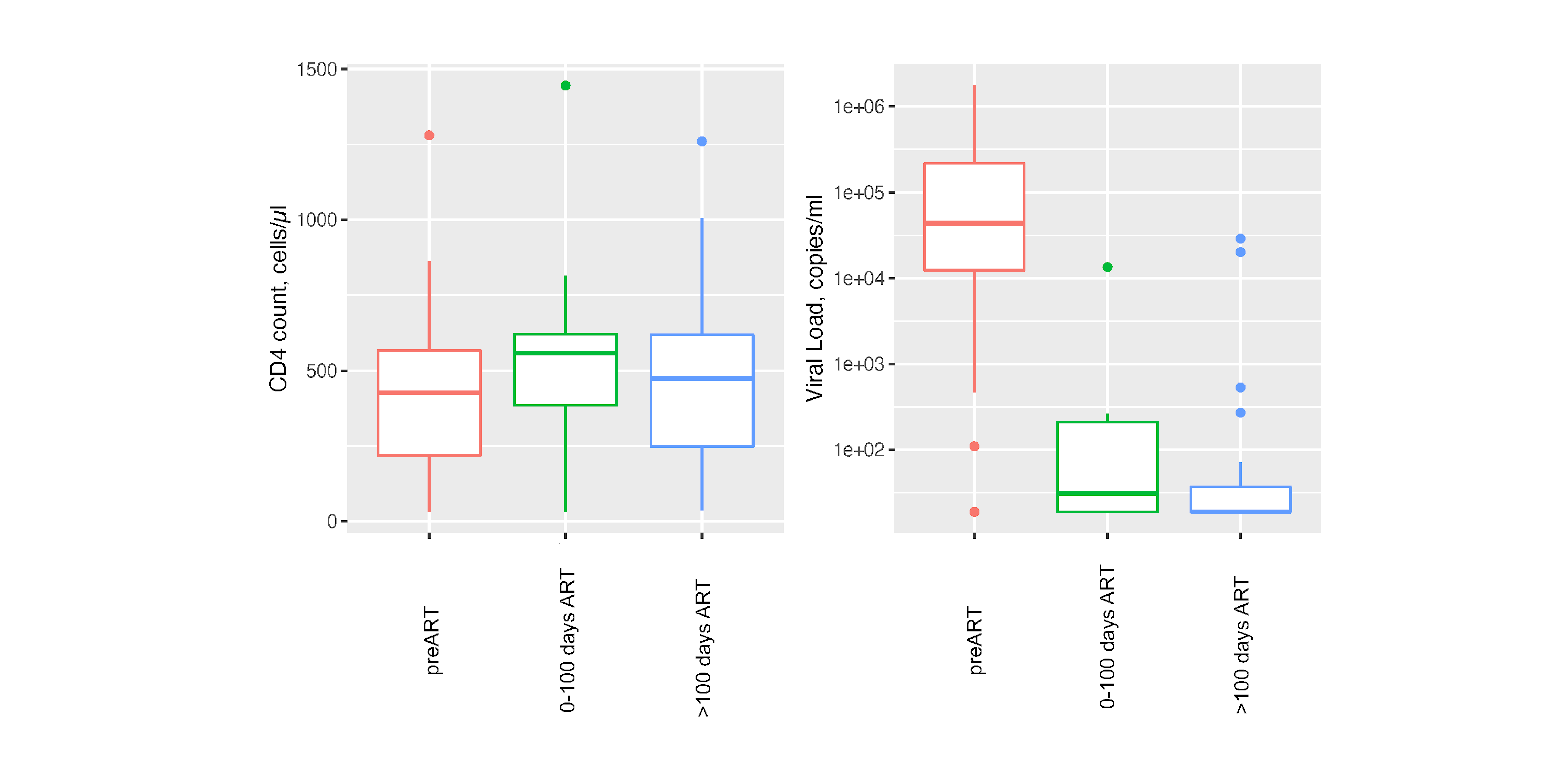

Supplement: FIG S1 [file mbio.00409-23-s0002.tif]
